# Physical Activity and Bone Vascularization: A Way to Explore in Bone Repair Context?

**DOI:** 10.3390/life11080783

**Published:** 2021-08-02

**Authors:** Rkia Wazzani, Stéphane Pallu, Céline Bourzac, Saïd Ahmaïdi, Hugues Portier, Christelle Jaffré

**Affiliations:** 1 Laboratoire APERE, Université de Picardie Jules Verne, CEDEX, F-80000 Amiens, France; rkia.wazzani@etud.u-picardie.fr (R.W.); said.ahmaidi@u-picardie.fr (S.A.); 2 Laboratoire B3OA, Université de Paris, CEDEX, F-75010 Paris, France; stephane.pallu@univ-orleans.fr (S.P.); c_bourzac@yahoo.fr (C.B.); hugues.portier@univ-orleans.fr (H.P.); 3 UFR Science & Technique, Université d’Orléans, CEDEX, F-45100 Orléans, France

**Keywords:** physical activity, bone vascularization, angiogenesis, VEGF, bone repair

## Abstract

Physical activity is widely recognized as a biotherapy by WHO in the fight and prevention of bone diseases such as osteoporosis. It reduces the risk of disabling fractures associated with many comorbidities, and whose repair is a major public health and economic issue. Bone tissue is a dynamic supportive tissue that reshapes itself according to the mechanical stresses to which it is exposed. Physical exercise is recognized as a key factor for bone health. However, the effects of exercise on bone quality depend on exercise protocols, duration, intensity, and frequency. Today, the effects of different exercise modalities on capillary bone vascularization, bone blood flow, and bone angiogenesis remain poorly understood and unclear. As vascularization is an integral part of bone repair process, the analysis of the preventive and/or curative effects of physical exercise is currently very undeveloped. Angiogenesis–osteogenesis coupling may constitute a new way for understanding the role of physical activity, especially in fracturing or in the integration of bone biomaterials. Thus, this review aimed to clarify the link between physical activities, vascularization, and bone repair.

## 1. Introduction

Physical activity is widely recognized as a biotherapy by WHO (World Health Organization) in the fight and prevention of bone diseases such as osteoporosis in older subjects [1]. Physical exercise-induced improvement in bone mineral density (BMD) has also been observed in young patients treated with glucocorticoids [2] or suffering from excessive alcohol consumption [3], both of these substances affecting bone viability. Exercise reduces the risk of disabling fractures associated with many comorbidities, and whose repair is a major public health and economic issue. It is classically accepted that physical activity in this context helps maintaining or increasing bone mass through mechanical stimulation [4,5] and systemic factors’ (hormones, growth factors, etc.) production [6]. Relevant mechanisms may also include an increase in vascularization within bone tissue, such as reported in previous studies that tend to show a link between the processes of angiogenesis, vascularization foundation, and osteogenesis involved in bone tissue formation [7].

**Hypothesis:**  *As physical activity improves vascularization, it also improves bone healing.*

Thus, physical activity could promote the link between angiogenesis and osteogenesis through both the compressions generated and the growth factors produced. The effects of exercise on these parameters require, however, further investigations [7]. Finally, in the treatment of osteoporosis, particular attention should be paid to the effects of physical activity on bone defect or fracture repair processes that take place in the osteoporotic bone. Although vascularization is highly involved in the bone repair process, studies demonstrating the preventive/curative effects of physical activity on bone repair have not investigated the contribution of vascularization to these effects specifically. Such studies are probably hindered, at least in humans, by the complexity of the approach and visualization of the results. That being said, the role of physical activity on the vascularization and repair of skeletal muscles or cardiovascular tissues is no longer to be demonstrated, which provides sound grounds for hypotheses to explore in a bone repair context.

The present work is at a crossroads: (1) It outlines the role of physical activity in the link between angiogenesis and osteogenesis in in vitro studies and pre-clinical animal model studies and (2) it proposes to expand these findings to the possible role of physical activity in the repair of non-critical-sized bone defects.

## 2. Physical Activities and Their Effects on Angiogenesis and Osteogenesis

It has been proven that moderate to vigorous physical activity improves health [5,8,9]. Increased attention is being paid to the effects of physical exercise on the skeleton and the whole body. Moderate to intense training or running exercise is known to improve body composition in humans [10]. In addition, intermittent (interval or fractionated) high-intensity training improves aerobic capacity and oxygen consumption in individuals [11]. Physical exercise is also characterized by its anti-inflammatory effect [12]. For bone, numerous studies have focused on the effects of different physical exercise modalities on bone quality [13] and on the bone parameters determining this quality: geometric parameters (cortical bone thickness, bone size, femoral neck geometry), architectural parameters (cortical porosity, trabeculae shape), and tissue properties (mineralization, cell density, osteocyte networks) [14]. Bone tissue is known for its sensitivity to mechanical stresses and its ability to support these [15]. Consequently, it is capable of adapting to these constraints in order to maintain its metabolic and phosphocalcic balance and constant remodeling [16]. The theories about bone response to stress include the production of multiple mechanical loads, along with the circulation of interstitial fluid in the lacuna-canalicular system [16]. To respond to mechanical strains and strengthen bone tissue, as a dynamic tissue, requires a combination of elements, including angiogenesis, which, besides, proved to be essential in bone repair and regeneration [17]. To date, literature data on the effects of exercise on angiogenic–osteogenic responses are scarce. Moreover, not to even mention the effects of physical activity, very little is known about the cellular and molecular mechanisms related to angiogenesis–osteogenesis coupling [18]. Yet, understanding these mechanisms should enable identifying new therapeutic approaches against bone diseases [19]. Indeed, the increased vascularization of bone tissue and the great impact induced by physical exercise on this tissue makes bone angiogenesis a target of choice for studies aiming at the prevention of bone loss diseases via non-pharmaceutical pathways. The works investigating the effect of physical exercise on bone vascular status have focused on different sets of angiogenic and vascular parameters including blood flow, endothelial function, arterial properties, etc. (Table 1).

The study by Matsuzaki et al. (2007), for instance, showed that mechanical loading of the anterior limbs from rats produces a rapid increase in periosteal vascularization associated with an increase in bone capital [29]. This indicates that angiogenesis and osteogenesis are spatially coordinated in the bone response to mechanical stimulation. A previous study, evaluating moderate (3 weeks) and intensive (7 weeks) treadmill training programs in rats and mice, demonstrated vascular adaptations in long bones, especially a significantly larger circulating blood volume in trained animals compared to respective results in sedentary animals [24]. Moreover, in trained animals, the circulating blood volume in femurs was higher than in tibias or humeri. This result may be explained by differences in mechanical stress intensities sustained by these different bones during exercise [24].

Stabley et al. (2014) showed an increase in generalized hyperemia in the posterior limbs and bone marrow during and after physical training [20]. Therefore, the authors suggested that increased bone and bone marrow blood flow during and after physical training leads to an increase in pressure in different bone regions and promotes bone interstitial fluid flow from the high-pressure area to the low-pressure area, thereby supporting osteogenesis and bone integrity [7,20]. This support of osteogenesis by osteoblastic formation is even accentuated in bone fracture. Indeed, an increase in bone blood flow with greater vascularization in the callus area was observed in rats trained 4 weeks after sustaining a femur osteotomy [27]. In contrast, short-term exhaustive exercise on a treadmill had no effect on total blood flow in the femoral bone marrow and only increased blood flow in the metaphyseal regions relative to the diaphyseal region [30]. The duration and intensity of the exercise are factors that affect angiogenesis and, thus, potentially bone repair.

In 2004, Yao et al. showed for the first time that, compared to sedentariness, only 2 weeks of running exercise in rats increased the number of vessels in the tibial proximal metaphysis by 20 folds and induced a positive regulation of VEGF receptor mRNA [26]. However, when an anti-VEGF treatment was used, exercise-induced adaptations (especially increases in bone mineral density, trabecular bone volume, and vessel density) were mitigated and an increase in osteoclastic surfaces was observed [26]. Taken together, these results provided evidence that angiogenesis and osteogenesis are tightly related.

Additionally, in rats, Dominguez et al. (2010) showed that the treadmill exercise-induced increase in bone blood flow and the NOS signaling pathway were associated with increased trabecular bone volume in the distal femur from young and old animals by 34% and 26%, respectively, compared to respective results obtained in sedentary animals [21]. In the same vein, a recent study conducted in ovariectomized rats demonstrated that, in addition to trabecular bone integrity, femoral angiogenesis was significantly improved in running rats. This was associated with improved femoral artery vasodilatation in running rats [25]. Taken together, these results confirm that bone angiogenesis is closely linked to exercise-induced osteogenesis and is essential for the bone gain sought by exercise, both in healthy [26], aging, or pathological contexts (osteoporosis) [21,25]. This coupling is generally explained by the increased NO-dependent vasodilation induced by physical training, associated with increased production and number of circulating endothelial progenitor cells (EPCs) in the bony vascular system in animals [28]. This vasodilatation promotes blood supply and angiogenesis, which, in turn, facilitates osteogenesis at different bone sites.

Apart from treadmill running, the effects of swimming on bone modeling have also been studied. They remain still questionable though [22]. Regarding bone vascular status, however, Viboolvorakul et al. (2009) demonstrated that 8 weeks of swimming could improve age-induced bone capillary vascular alterations in rats [22]. Similarly, Park et al. (2014) showed that swimming for 8 weeks in ovariectomized rats suppressed peripheral artery endothelial dysfunction but did not prevent bone loss [23]. In contrast, voluntary climbing exercise resulted in significant improvements in both endothelial function and bone mass in the postmenopausal rat model [23].

Until today, research works describing the effects of physical exercise on bone vascular parameters in humans have been very rare. A few years ago, the value of activity resumption (re-education) to regain the radial artery distensibility altered by fractured-arm immobilization in humans was demonstrated [31]. To our knowledge, however, there are no studies associating angiogenesis with osteogenesis and exercise modeling in humans or explaining the cellular and molecular mechanisms involved. Nevertheless, the positive effects of exercise on global vascular status have been well demonstrated by several studies. Laufs et al. (2004) observed a significant increase in circulating EPCs after bicycle ergometer training (Table 1) with a reduced apoptosis rate compared to respective results obtained pre-training [28]. In 2014, Ross et al. also demonstrated an increase in EPCs and angiogenic factors (VEGF in particular) for the first time in response to a resistance exercise [32]. Similarly, Adams et al. (2004) noted in patients with symptomatic coronary artery disease a significant increase in circulating EPCs in the peripheral blood, preceded by an increase in plasmatic VEGF in response to exercise-induced myocardial ischemia [33]. With this result, the authors confirmed that VEGF plays an important role in the migration of EPCs from the bone marrow to the peripheral blood. In 2005, Laufs et al. studied the effect of physical exercise on circulating EPC in healthy subjects this time [34]. Two different running protocols were evaluated. The results showed that 30 min of moderate-intensity running increased the number of circulating EPCs in contrast to the 10-min, moderate, short-term run [34]. Overall, other studies investigating the effects of physical exercise on EPCs showed that intense physical exercise significantly increases both the number of EPCs and the release and mobilization of EPCs in the peripheral blood [35,36].

In general, the migration of EPCs from bone marrow to peripheral sites depends on many factors, but the mechanism of this mobilization and differentiation is still not well defined. Studies suggest, however, that these cells can promote local angiogenesis by secreting angiogenic factors via a paracrine pathway [37]. It should also be noted that the number of EPCs is strongly influenced by factors such as drugs, growth factors, and, of course, physical exercise [37]. All these elements suggest that EPCs are an important actor to study in the scope of the vascular effects of physical exercise in humans.

**Figure 1:**
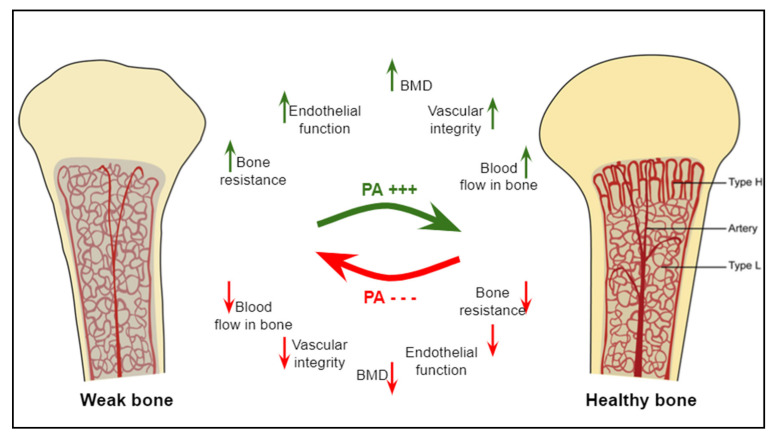
The effects of physical activity or mechanical loading on bone parameters. The benefits of physical activity on bones, their microarchitecture, and microvascularity are multiple, including improved BMD, bone strength, bone blood flow, and the vascular integrity. The absence of physical activity deteriorates these bone and vascular parameters. Columnar type-H and sinusoidal type-L vessels are bone microvessels located in the metaphysis and in the diaphysis, respectively. Modified from [38].

Overall, physical exercise is classically known as a bone anabolic agent [32]. Additionally, exercise is also known to improve bone angiogenic adaptations through the regulation of key angiogenic mediators (VEGF, FGF, etc.) [7]. These adaptations in the vascular system precede those occurring in bone in response to mechanical stimulations. Therefore, a better understanding of the angiogenic and osteogenic mechanisms induced by different types of physical exercise should allow the identification of new non-pharmacological strategies to prevent bone fragility (Figure 1).

## 3. Some Growth and Transcriptional Factors Involved in Osteogenesis/Angiogenesis Induced by Physical Activity

Bone remodeling in repair process depends on strong interactions between osteoblasts and other cells present within the bone microenvironment, especially vascular endothelial cells [39,40]. Some research teams have previously demonstrated the in vitro link between osteogenesis and angiogenesis [41]. Villars et al. (2002) demonstrated, by dye coupling assay with Lucifer yellow, a functional coupling between human umbilical vein endothelial cells (HUVEC) and human bone marrow stromal cells (HBMSC). They showed by immunocytochemistry that the connexin 43 (C × 43), a gap junction protein, was expressed not only in HBMSC but also in the endothelial cell network, and that these two cell types communicated through a gap junctional channel constituted at least by C × 43 [41]. Moreover, they also showed that a 3-day to 3-week HUVEC (Human osteoprogenitor cells (HOP) co-culture) stimulated HOP differentiation and mineralization. Finally, they demonstrated by reverse-transcription, real-time quantitative PCR that in such a co-culture model, an up-regulation of the ALP expression in the co-cultured HOP was observed within the first 48 h [42].

Many factors are involved in this angiogenesis–osteogenesis coupling, but, to date, only few of them have been studied in the physical activity or mechanical loading context. We, therefore, limited the scope of this review to the principal factors analyzed, such as VEGF, HIF-1, eNOS, FGFs, BMPs, MMPs, and Notch ligands [43,44].

VEGF: The most studied factors were the vascular endothelial growth factors VEGFs, which are major regulators of angiogenesis and act in endothelial cell proliferation, migration, and activation [45,46]. Due to angiogenesis–osteogenesis coupling, VEGFs also influence skeletal development and postnatal bone repair. VEGFs come in five dimeric polypeptides forms: VEGFA (the prototype), VEGFB, VEGFC, VEGFD, and placenta growth factors (PlGF). They bind to VEGF receptors (1, 2, and 3), Nrp1, and Nrp2, and become activated upon ligand binding [47,48]. Studies have previously indicated that VEGF expression increases in various tissues (brain, lung, skeletal muscle, for instance) with different exercise trainings [49,50,51,52]. In cancellous bone, Yao et al. (2004) found a significant increase in VEGF and VEGF-r1 mRNA expressions after 10 days of running at 60% VO_2_max; these expressions were maintained up to 5 weeks after training was stopped [26]. It was accompanied in the tibial metaphysis by an increase in both the number of intramedullary vessels and cancellous bone formation as well as a decrease in resorption. It was also demonstrated in an in vitro model of osteocyte physical damage that mechanical stimuli had an additive effect on VEGF mRNA expression and its concentration in culture media [53]. Taken together, these few studies provide evidence of a positive effect of physical activity or mechanical strains on angiogenesis, via VEGF.

HIF-1α: Physical activity is associated with reduced oxygen levels in numerous tissues (skeletal muscle, cardiac muscle, bone, etc.) and improve cellular response to hypoxia by the production of hypoxia-inducible factor-1 (HIF-1). HIFs constitute a transcriptional regulator, which supports neo-angiogenesis [54] and regulates the expression of vascular endothelial growth factor (VEGF) [55]. HIFs are heterodimers, composed of an oxygen-regulated α-subunit and an oxygen-independent β-subunit. There are three HIF-α family proteins identified in humans: HIF-1α, -2α, and -3α [56]. HIF-1α plays an important role in coupling angiogenesis and osteogenesis, particularly in skeletal healing and development [57,58,59]. Physical training could increase HIFs, especially HIF-1α, in skeletal muscle [60,61]. Using a mechanical loading protocol, consisting of either damaging or non-damaging axial compression of the right forelimb in mice, Tomlinson and Silva (2015) showed that HIF-1α activity depended on loading characteristics: HIF-1α was pro-osteogenic for woven bone formation under damaging loading conditions but anti-osteogenic for lamellar bone formation under non-damaging mechanical loading [62]. In another study [57], the authors showed that activation of the transcription factor HIF-1α is a primary response to bone mechanical loading and that it would function in osteoblasts as a negative regulator of load-induced bone formation. Taken together, these studies suggest that the angiogenic response to mechanical loading is mediated through HIF-1α expression and that HIF-1α may alter bone formation and repair.

eNOS: Other factors could be activated by oxygen deficit. Nitric oxide (NO) is a free radical regulating bone cell function. The endothelial isoform of nitric oxide synthase (eNOS) is constitutively expressed in bone, whereas inducible NOS is only expressed in response to inflammatory stimuli. The eNOS isoform seems to play a key role in regulating osteoblast activity and bone formation [63]. It has been shown that exercise training promotes eNOS production in coronary endothelial function [64] and left ventricular [65], and, by enhancing acetylcholine-induced femoral artery vasodilation and bone angiogenesis, improves the blood supply of bone, thus facilitating osteogenesis, in osteoporotic sites, for instance [25].

FGFs: Fibroblast growth factors are growth factors that play a role in cell proliferation, migration, and differentiation in various organs, including bone. The FGF family comprises three subfamilies known as canonical, hormone-like, and intracellular. The roles of canonical and hormone-like FGFs have been characterized in bone differentiation [66]. Concerning bone vascularization, FGF-2 administration may contribute to the treatment of ischemic osteonecrosis [67]. Similarly, an absorbable collagen sponge with FGF-2 increased blood vessel and bone formation in rat calvarial critical-sized bone defects [68]. These studies confirm the role of FGF-2 in bone vascularization, in particular, during bone defect healing and, therefore, its involvement in bone repair processes. To our knowledge, there are no data about the effects of physical activity on FGFs in bone tissue. Previous studies have, however, identified their presence in muscles [69], tendons, and ligaments [70] following various exercise protocols, whereas they were absent in control subjects. Similar results could, therefore, be expected in bone tissue.

BMPs: Bone Morphogenetic Proteins (BMPs) constitute the largest subdivision of the transforming growth factor- β (TGF- β) family of ligands. They initiate a biological cascade that involves multiple cell types and signaling events and culminates in the production of functional bone tissue [71]. For example, they initiate osteoblastic differentiation [72]. BMP signaling is central in endothelial cells of blood vessels [73]. Of note, BMP-7 mRNA and protein expression have been shown to increase in muscles with endurance training and gradual exercise [69,74]. In bone, the effects of training or exercise remain to be confirmed, even though Siamwala et al. (2015) highlighted, in a review on microgravity, the importance of physical activity on BMPs’ expression in preventing bone loss [75].

MMPs: Matrix mettaloproteinases (MMPs), a family of endopeptidases (Zn^+2^ dependent), mediate various physiological processes by digesting components of the extracellular matrix [76], including osteoblast/osteocyte differentiation, bone formation, solubilization of the osteoid during bone resorption, osteoclast recruitment and migration, angiogenesis, and as a coupling factor in bone remodeling under physiological conditions [77,78]. Numerous studies have shown that physical activity and mechanical stresses are accompanied by a stimulation of MMPs’ synthesis (−2, −9, −13) and may play a role in angiogenesis (bone, muscles, vertebral disk, etc.) [79,80].

Notch: Notch signaling pathway contributes to regulate cell–cell interactions. In bone tissue, the activation of Notch signaling leads to enhanced osteogenesis and angiogenesis [19]. Many studies have indicated that physical training or mechanical loading stimulated Notch signaling pathway in different tissues [19]. However, in bone tissue, data are missing, especially in the presence of physical activity or mechanical loading.

## 4. Vascularization and Bone Repair

Few studies have focused on the impact and evolution of vascularization in bone repair. Fractures are the most common large-organ, traumatic injuries to humans with, however, a very high regeneration capacity [81,82]. As for the development of bone, bone fracture healing is a complex multistep process. It starts with the formation of a hematoma around the fractured region of the bone and involves cytokine-secreting inflammatory cells for forming a fibrinous clot [17,83]. In the early callus, mesenchymal stem cells differentiate into chondrocytes, which further promote vascularization and bone formation through the secretion of several proteins (BMPs, MMP-13, alkaline phosphatase, VEGF, and placental growth factor). Chondrocytes in the callus also release anti-angiogenic factors, which limit blood vessel growth [84]. Additionally, the vascular network is essential for proper bone formation and functional bone restoration [17,18,85,86,87,88,89]. If blood flow is impaired, bone healing and repair may be delayed [90]. Indeed, it is admitted that the presence of blood vessels is critical for a successful bone regeneration [91,92]. The lack of blood supply induces local hypoxia, which may be maintained by subsequent inflammation [93]. The observed pO2 (partial pressure of oxygen) post-fracture is 1–3% [88]. This trauma-induced hypoxia causes endothelial cells (ECs) to upregulate bone morphogenic proteins (BMPs; i.e., BMP-2) to promote osteogenesis [17,89]. The hypoxia-induced transcription factor (HIF)-α pathway, activated in hypoxia, is considered a key mechanism for coupling bone growth to angiogenesis via increased expression of VEGF, the major vascular growth factor expressed by hypoxic osteoblasts [88]. In mice, where HIFα is overexpressed, the bones are highly vascularized [94]. In addition, the placental growth factor (PlGF, a VEGF homologue), which acts through the VEGF receptor, also appears to play a significant role in fracture healing [93]. VEGF stimulates the regrowth of blood vessels into the injury site, so that oxygen and nutrient levels return to normal values [88]. The general pattern for bone healing consists in a recruitment of osteoclasts in the early hypoxic phase, while revascularization progressively favors osteoblast function (proliferation, differentiation, and bone formation). Osteoblast precursors could also move into fractured bones concomitant with the invasion of the blood vessels [83]. Additionally, osteoblasts accumulate HIF-1a, leading to VEGF-A production, which further enhances angiocrine BMP production [84,89,94,95,96]. As for embryonic bone development, fibroblast growth factors (FGFs) are also required for bone repair. In Behr et al.’s study (2011), mice with heterozygous FGF9 loss, neovascularization, and cortical repair were reduced, in part due to reduced VEGF-A, and after a combined administration of FGF9 and VEGF-A the defect was significantly reduced [97]. Further, Kigami et al. (2013) showed that FGF-2 enhanced angiogenesis in rat calvarial critical-sized bone defects [68]. Even if knowledge has yet to be widely developed, other molecular processes are also involved. For example, some works highlight the central roles of Notch signaling in bone endothelium and its regulation by blood flow, which is relevant for age-related bone loss and, potentially, for therapeutic approaches aiming at the maintenance or restoration of bone mass. Notch pathway genes are highly expressed in arterial ECs, which are exposed to high flow rates and shear stress [17,34]. Based on the principle that active blood supply is essential for callus formation during fracture healing and repair [86], methods to artificially increase blood flow during early steps of repair are used as treatment [84]. However, the relationship between angiogenesis and bone repair may be more nuanced. This suggests a more complex balance or interplay between pro- and anti-angiogenic factors in the healing process.

Recently, it has been shown that preventive moderate continuous running-exercise conditioning could improve the healing of non-critical-sized bone defects in male Wistar rats [98]. In another study, mice were put in cages that were supplied with running wheels after a cranial bone window. Spontaneous physical activity promoted angiogenesis during bone repair in this model over a time period of 21 days [99].

## 5. Conclusions

In conclusion, it is now well established in the literature the importance of good bone vasculature for the preservation of bone integrity. Furthermore, different physical exercise modalities have been identified by their angiogenic and osteogenic improvements in humans and animals. However, the mechanisms coupling angiogenesis and osteogenesis in response to an exercise modality are still poorly understood. This provides an opportunity for new descriptive but also analytical scientific studies to shed light.

Finally, a deeper knowledge of the mechanisms involving physical exercise used in preventive or curative mode in the revascularization of bone could open new fields of use and investigation both in the repair of fractures and as a complement in the use of bio-materials for defects of critical size.

## Figures and Tables

**Table 1:** Effects of various exercises on vascular parameters. The effects are expressed in degree of positivity (+ to + + +).

Type of Exercise	Duration	Species	Site	Parameters	Effects	References
Treadmill walking	10–12 weeks	Rat	Femur Tibia Fibula	Blood flow and vascular conductance	+ + +	[20]
Treadmill exercise	10–12 weeks	Rat	Femur	Bone marrow blood flow	+	[21]
				Vasodilatation response	+	
				Endothelium signaling pathways	+	
Swimming	8 weeks	Rat	Femur	Bone microvascularity Bone capillary vascularity	+ + +	[22]
Climbing resistance exercise	8 weeks	Rat	Aorta	Endothelin-1 concentration Expression of the endothelial nitric oxide (eNOS) protein	+ + +	[23]
Swimming	8 weeks	Rat	Aorta	Endothelin-1 concentration Expression eNOS protein	+ + +	[23]
Treadmill running	7 weeks 3 weeks	Rat Mouse	Humerus Femur Tibia	Vascular adaptations Circulating red cell volume Circulating blood volume	+ + +	[24]
Treadmill exercise	6 weeks	Rat	Femur	Diameter and volume of blood vessels Expression of eNOS in femoral vessels	+ + +	[25]
Treadmill running	5 weeks	Rat	Tibia	Number of vessels in the proximal metaphysis VEGF receptor 1 mRNA	+ + +	[26]
Treadmill running	4 weeks	Rat	Femur	Total bone, proximal, diaphyseal, callus, and muscle blood flows	+ + +	[27]
Treadmill running	3 weeks	Mouse	Bone marrow	Numbers of circulating EPCs EPCs in the bone marrow VEGF serum level	+ + +	[28]
Bicycle ergometer training	4 weeks	Human	Blood	Numbers of circulating EPCs	+ + +	[28]

## Data Availability

Not applicable.
